# Fabrication and Biomedical Application of Alginate Composite Hydrogels in Bone Tissue Engineering: A Review

**DOI:** 10.3390/ijms25147810

**Published:** 2024-07-17

**Authors:** Xiuqiong Chen, Ting Wu, Yanan Bu, Huiqiong Yan, Qiang Lin

**Affiliations:** 1 Key Laboratory of Tropical Medicinal Resource Chemistry of Ministry of Education, College of Chemistry and Chemical Engineering, Hainan Normal University, Haikou 571158, China; chenxiuqiongedu@163.com (X.C.); wt09250916@163.com (T.W.); buynedu@163.com (Y.B.); linqianggroup@163.com (Q.L.); 2 Key Laboratory of Water Pollution Treatment & Resource Reuse of Hainan Province, College of Chemistry and Chemical Engineering, Hainan Normal University, Haikou 571158, China; 3 Key Laboratory of Natural Polymer Functional Material of Haikou City, College of Chemistry and Chemical Engineering, Hainan Normal University, Haikou 571158, China

**Keywords:** alginate, biomacromolecule, composite hydrogels, functional biomedical scaffold, bone tissue engineering

## Abstract

Nowadays, as a result of the frequent occurrence of accidental injuries and traumas such as bone damage, the number of people causing bone injuries or fractures is increasing around the world. The design and fabrication of ideal bone tissue engineering (BTE) materials have become a research hotspot in the scientific community, and thus provide a novel path for the treatment of bone diseases. Among the materials used to construct scaffolds in BTE, including metals, bioceramics, bioglasses, biomacromolecules, synthetic organic polymers, etc., natural biopolymers have more advantages against them because they can interact with cells well, causing natural polymers to be widely studied and applied in the field of BTE. In particular, alginate has the advantages of excellent biocompatibility, good biodegradability, non-immunogenicity, non-toxicity, wide sources, low price, and easy gelation, enabling itself to be widely used as a biomaterial. However, pure alginate hydrogel as a BTE scaffold material still has many shortcomings, such as insufficient mechanical properties, easy disintegration of materials in physiological environments, and lack of cell-specific recognition sites, which severely limits its clinical application in BTE. In order to overcome the defects of single alginate hydrogels, researchers prepared alginate composite hydrogels by adding one or more materials to the alginate matrix in a certain proportion to improve their bioapplicability. For this reason, this review will introduce in detail the methods for constructing alginate composite hydrogels, including alginate/polymer composite hydrogels, alginate/bioprotein or polypeptide composite hydrogels, alginate/bioceramic composite hydrogels, alginate/bioceramic composite hydrogels, and alginate/nanoclay composite hydrogels, as well as their biological application trends in BTE scaffold materials, and look forward to their future research direction. These alginate composite hydrogel scaffolds exhibit both unexceptionable mechanical and biochemical properties, which exhibit their high application value in bone tissue repair and regeneration, thus providing a theoretical basis for the development and sustainable application of alginate-based functional biomedical materials.

## 1. Introduction

Nowadays, with the rapid development of the world economy, the number of patients with bone injury and fracture caused by accidental injury shows an increasing trend [1]. There are about 15 million fracture cases in the world every year, and about 2.2 million bone transplantation operations are performed worldwide every year [2,3]. Although bone has the ability to self-heal and regenerate, when bone is severely damaged and exceeds the ability of the bone to self-heal and regenerate, the clinical treatment of bone transplantation is required. Currently, the clinical treatment approaches for bone defect or fracture include autologous bone grafting and allograft bone grafting. Autologous bone grafting is deemed the “gold criterion” for the treatment of bone defects. Autologous bone grafting refers to the transplantation of bone obtained from the patient’s own body to the site of bone shortcomings with the intent of repairing bone tissue shortcomings [4,5]. The advantage of autologous bone transplantation is that it avoids the occurrence of rejection and improves the survival rate of bone tissue, whereas its disadvantage is that it is limited by its own bone mass [6]. However, allogeneic bone transplantation overcomes the shortcomings of autogenous bone transplantation, but compared with autogenous bone transplantation, it has poorer bone healing outcomes and is associated with the occurrence of rejection reaction and the risk of disease transmission [7,8]. Therefore, both autogenous bone transplantation and allogeneic bone transplantation have their own shortcomings, which limits their wide application. Therefore, it is necessary to research and develop bone graft substitutes to make up for their shortcomings. Bone tissue engineering (BTE) is considered to be the most promising method for repairing or treating bone tissue defects. BTE scaffold materials provide a three-dimensional physiological environment for osteoblast adhesion, proliferation, and differentiation by simulating the physiological function and structure of the osteoblast extracellular matrix, so that bone cells and scaffold materials can cooperate to induce bone tissue repair and regeneration, thus achieving the purpose of repairing or treating bone.

Currently, the reported BTE scaffold materials include natural polymer, metal materials, synthetic organic polymer, bioceramic materials, and bioglass [9]. Among them, titanium (Ti) and its alloy are the most studied metal scaffold materials. Because of its good biocompatibility and excellent mechanical properties, it has been used in clinical practice as a bone replacement material. However, as scaffold materials, titanium and its alloys have defects such as poor biological activity, low bone conductivity, inability to degrade in the human body, and high price, which limits their wide application in bone tissue engineering [10]. Bioceramic materials, such as hydroxyapatite (HAP), possess the ability to guide bone formation as well as have good bone conductivity and biocompatibility due to their similar composition to the inorganic composition of bone. However, bioceramic scaffold materials have disadvantages, such as high brittleness and poor plasticity, which restricts their wide clinical application. There have been reports of synthetic organic polymer scaffolds made of polyethylene glycol (PEG), polyvinyl alcohol (PVA), polylactic acid (PLA), polyacrylamide (PAM), polylactic acid glycolic acid (PLGA), and polycaprolactone (PCL). Although the benefits of synthetic polymers include good plasticity, biodegradability, and biocompatibility, they lack biological activity, and some by-products released during the degradation process lead to local physiological environment acidification, which may cause tissue necrosis and other shortcomings, restricting the wide application of synthetic organic polymers in BTE scaffolds [9,11].

However, the ideal BTE scaffold material should have the following merits: (1) good biocompatibility, a suitable specific surface area, non-toxic effects, and a three-dimensional space structure; (2) excellent porosity and pore size that can satisfy the adhesion, proliferation, and differentiation of osteoblasts on scaffolds; (3) easy source and low price; (4) excellent mechanical properties; and (5) good biodegradability [12]. In contrast, natural biomacromolecules have excellent biocompatibility, biodegradability, non-toxicity, abundant sources, and low immunogenicity, which make them have unparalleled advantages in BTE scaffold materials [13,14]. Among many natural biomacromolecules, alginate has attracted much attention for its advantages, including non-toxicity, good biocompatibility, biodegradability, easy gelation, non-immunogenicity, wide source, and low price [15,16,17]. At comparatively mild pH values and temperatures, it can be crosslinked with bivalent calcium ions. The resulting alginate hydrogel that has an open network structure plays a coating and protective role in cells, which can promote the exchange of cellular nutrients and metabolites [18]. The previous research results confirmed that mouse preosteoblasts, human adipose stem cells, and bone marrow stromal cells can all survive in alginate hydrogel and form an extracellular matrix, serving as a foundation for the investigation of alginate hydrogel as a BTE matrix material [19,20]. However, it is challenging for hydrogel scaffolds made with a single alginate to satisfy every performance demand of the perfect scaffold. To overcome the defects of a single material, many researchers add one or more other materials to the alginate matrix in a certain proportion and as a way to prepare composite materials, so as to realize the complementary advantages of the materials [21,22]. For this reason, in order to develop ideal BTE scaffold materials with excellent alginate, this review will introduce in detail the methods of constructing alginate composite hydrogels, as well as their biological application trends in BTE scaffold materials, and look forward to their future research directions. The aim of this review is to establish a theoretical foundation for the development and sustainable application of alginate-based functional biomedical materials.

## 2. Alginate and Its Crosslinking Feature

### 2.1. Alginate Property

**Figure 1:**
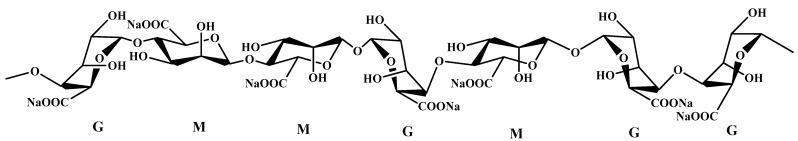
Molecular structure of sodium alginate for 1,4-β-D-mannouronic acid (M) and 1,4-α-L-gulonuronic acid (G).

Alginate is a widely used marine biomolecule, second only to cellulose in the world. It was first extracted from brown algae for scientific research by British chemist E.C. Stanford. Alginate includes potassium alginate, magnesium alginate, sodium alginate, and its corresponding ammonium salt and calcium salt, and the alginate extracted from seaweed is usually dominated by sodium alginate, while the source of sodium alginate is not only algae, but also some bacteria [23,24]. As a natural hydrophilic anionic polysaccharide, sodium alginate has a molecular formula of (C_6_H_7_O_6_Na)n, and its relative molar mass in the general market is in the range of 32,000~400,000 g/mol [25]. As depicted in Figure 1, sodium alginate is a linear natural polymer formed by 1,4-β-D-mannouronic acid (M) and 1,4-α-L-gulonuronic acid (G) connected by a 1–4 glucosidic bond in an alternating or block form, and the theoretical value of its basic structural unit molar mass is 198.11 g/mol [26,27]. As a natural biomacromolecule, it is not soluble in ether, chloroform, or ethanol, among other organic solvents, and is insoluble in ethanol solution with a mass fraction greater than 30% and acid solution with a pH < 3, but it can slowly dissolve in cold water to form a viscous liquid [25]. Furthermore, its solubility will also affect the normal operation of some chemical reactions of alginate, such as the oxidation reaction between alginate and a sodium periodate aqueous solution, which can only be carried out in an alginate solution with a mass fraction less than 4% [26]. These special properties create great challenges for the chemical modification of alginate. However, due to its non-toxic, biodegradable, good biocompatibility, and crosslinking properties, in 1983, the US Food and Drug Administration (FDA) authorized alginate for direct use as a material in the food industry [23]. Later, it was applied to biomedical engineering (such as drug delivery, tissue engineering, wound dressings, etc.), cosmetics, pharmaceuticals, packaging, and other fields, thus exhibiting great application potential through the reasonable design of alginate biomacromolecular materials [24,26,27].

Alginate has been used extensively in a variety of fields for its unique qualities, such as biocompatibility, biodegradability, water solubility, low immunogenicity, versatility, relatively low cost, thickening property, and gellability [17]. For example, alginate is widely used in the food field, where it is considered a crucial source of dietary fiber. To preserve food quality and flavor, edible films and coatings made of alginate can be used in food packaging. In addition, alginate’s gelling and thickening properties make it suitable as an additive for food, cosmetics, and paints [28,29]. Similarly, the carboxyl and hydroxyl functional groups found in alginate’s molecular skeleton, as well as its high water-absorption properties, result in alginate's extensive use in environment-related fields. It has shown great promise in capturing chemicals from pollution sources [30]. In addition, alginate can also be combined with other natural polymers (such as anionic or cationic polysaccharides, proteins, etc.) and synthetic polymers, organic or inorganic fillers, and bioactive substances to be applied in different fields [31]. Of all these applications, the BTE scaffold is the most important. In fact, the unique properties of alginate make it an extremely promising biomaterial that can serve a multitude of uses in basically any form (i.e., hydrogels, films, nanofibers, and gel microspheres), shape, and size. For example, alginate has been extensively studied in the last decade as a scaffold material for BTE applications [32].

### 2.2. Preparation of Alginate Hydrogels

**Figure 2:**
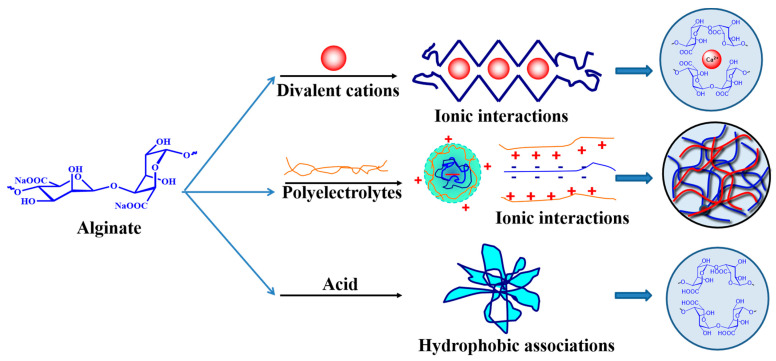
Schematic diagram of physically crosslinked alginate hydrogels.

Alginate is commonly used in the form of hydrogels in the biomedical domains of tissue engineering, drug delivery, and wound dressings. A hydrogel is a kind of three-dimensional (3D) scaffold material with a chemically or physically crosslinked structure swelling, but insoluble, in water. Because of its good hydrophilicity, biocompatibility, and degradability, and its mechanical properties and structure, which are very similar to the extracellular matrix, it can provide an ideal 3D microenvironment for cell culture [15]. Chemical and physical crosslinking are the most typical methods to form alginate hydrogels, and the type of crosslinking, crosslinking density, polymer molecular weight, and chemical composition completely determine the hydrogels’ chemical and physical characteristics [33,34]. According to the different crosslinking methods, physically and chemically crosslinked alginate hydrogels are the two categories into which they can be classified. Physical crosslinking is the most common method for preparing BTE scaffolders, which can be divided into ion crosslinking, polyelectrolyte crosslinking, and alginate acid crosslinking, as shown in Figure 2 [12,35].

#### 2.2.1. Ion Crosslinking

Ion crosslinking is the most common method for fabricating alginate hydrogels, because alginate interacts with divalent cations, such as Fe^2+^ or Mg^2+^, Ca^2+^, Sr^2+^, and Ba^2+^, to create alginate hydrogels in mild conditions [36]. The reason for the formation of hydrogels is that the interaction between the divalent cation and the α-L-guluronic acid (G) structural unit in alginate forms a strong and stable three-dimensional network structure, while the interaction between it and the β-D-mannouronic acid (M) structural unit forms a weak network structure, and finally the coordination between the divalent cation and four carboxyl groups forms an egg-box structure [25,37] (as shown in Figure 2). In general, bivalent cation Ca^2+^ is commonly used for ion crosslinking with alginate, which is due to the ion exchange process of alginate hydrogels in the medium, so that the bivalent cation crosslinked in the hydrogels can be dispersed into the surrounding medium. However, these bivalent cations, such as Ba^2+^ and Sr^2+^, have slight toxicity, which limits their application in BTE scaffold materials [38,39,40,41]. On the contrary, Ca^2+^ itself is non-toxic, so crosslinking agents containing Ca^2+^ ions, such as calcium chloride (CaCl_2_), calcium carbonate (CaCO_3_), calcium sulfate (CaSO_4_), hydroxyapatite (HAP), and calcium phosphate (Ca_3_(PO_4_)_2_), are commonly used to crosslink alginate to prepare alginate hydrogels [42]. In addition, compared with other hydrogels, the hydrogels formed by alginate and calcium ions also have the advantage of thermal irreversibility, which are able to have coating and protective effects on cells [15].

#### 2.2.2. Polyelectrolyte Crosslinking

The polyelectrolyte crosslinking of alginate refers to the creation of polyelectrolyte complexes (PECs) by the electrostatic interplay between polyelectrolytes and opposite charges (generally polycationic electrolytes) and polyanionic alginate [43]. Commonly used polycationic electrolytes include type A gelatin, polylysine, chitosan, polyethylenimine, and poly (L-ornithine) [44,45,46]. Chitosan, due to its advantages of good biocompatibility and abundant sources, is often reported to be used to prepare alginate/chitosan polyelectrolyte composite hydrogel scaffolds. For example, Yan et al. [47] used alginate, chitosan, gelatin, and other substances to prepare composite hydrogels by the layer-by-layer assembly method through electrostatic action as BTE scaffold materials. The outcomes demonstrated the excellent biocompatibility of the composite scaffold as well as its capacity to encourage cell adhesion, proliferation, and differentiation. Additionally, chitosan quaternary ammonium salt, polymer-grafted chitosan, and carboxymethyl chitosan may also be utilized to create polyelectrolyte hydrogels based on alginate, which provide them additional useful qualities and uses [48,49,50]. In addition, it is possible to develop composite hydrogel scaffolds in a novel way because the structural units in alginate have no effect on the mechanical properties of polyelectrolyte crosslinked composite hydrogels.

#### 2.2.3. Hydrophobic Association Crosslinking

If the alginate solution’s pH is quickly lowered by adding acid directly, an alginic acid hydrogel based on hydrophobic associations will be produced [51]. When the pH of the water solubility of alginate is adjusted below the pKa value of the monomer, a sol-gel transformation will occur, and the formation of this hydrogel depends on intermolecular hydrophobic associations and hydrogen bonding [52]. It has been reported that acid crosslinked hydrogels are currently prepared by adding lactones (e.g., D-(+)-gluconate δ-lactones) to an alginate solution for slow proton release or by the proton replacement of calcium alginate hydrogels into alginate acid hydrogels [52,53].

#### 2.2.4. Chemical Crosslinking

The chemical crosslinking of alginate is actually a three-dimensional network structure that is connected between polymer molecular chains through the covalent bonding of crosslinking agents. Because there are a lot of active groups, like -OH and -COOH, in the alginate molecular chain, these groups can be combined with glutaraldehyde, epichlorohydrin, diamine, borax, and other chemical crosslinking agents to prepare alginate composite hydrogels [54]. The variety and adaptability of these hydrogels are enhanced by the fact that alginate can also be crosslinked with other macromolecules through different kinds of reactions [55]. Desai and associates [56] produced a particular kind of alginate-based click hydrogel by means of a physiological Diels–Alder cyclo-addition reaction between tetrazine-modified alginate (Alg-T) and norbornene-modified alginate (Alg-N). One could modify the swelling and mechanical properties of the resulting hydrogels by adjusting the polymer concentration and stoichiometry of the functional groups where the click reaction took place. Although covalent crosslinking has been extensively researched as a means of enhancing the chemical and physical characteristics of alginate hydrogels, it is not recommended for use in the biomedical field due to the biological toxicity of certain crosslinking agents.

### 2.3. Mechanical Properties of Alginate Hydrogels

It is possible to modify the mechanical properties of alginate hydrogels by adjusting the polymer’s concentration in solution, as well as the number and order of G and M monomers [57]. Hydrogels with higher mechanical stiffness and strength are typically produced by alginates with a higher G content than those with a higher M content. The number density of physical crosslinks between chains—which are made possible by the presence of cations—determines the elastic modulus. Alginate hydrogels that gel more slowly have larger moduli and, consequently, more structural homogeneity than those that gel more quickly. Furthermore, lengthening the alginate chain or raising the alginate concentration in a precursor solution are common ways to enhance the mechanical characteristics of alginate hydrogels [58,59]. It is challenging to handle and produce homogenous hydrogels using these methods because they typically require a very viscous precursor alginate solution. Alginate hydrogels can be made significantly stiffer by adding more strong covalent crosslinks, but this usually causes the hydrogel to become brittle and lose strength [60]. To create alginate hydrogels that are mechanically reinforced, strong, stiff reinforcements like fibers and particles are frequently combined with the alginate solution.

### 2.4. Rheological Properties of Alginate Hydrogels

Since rheology is fast, sensitive, requires small sample sizes, and reveals architectural differences like molecular weight, degree of crosslinking, glass transition proximity, and structural homogeneity/heterogeneity, it is a suitable method for characterizing a hydrogel's mechanical properties [61]. When preparing hydrogels, the primary rheological properties of alginate-based hydrogels are crucial design factors. A novel approach to the rheological testing of alginate-based hydrogels has been evaluated to ensure consistent and dependable outcomes concerning storage moduli and loss. The approach takes into consideration how morphology affects rheological characteristics. By using the suggested methodology, information regarding the hydrogels’ degree of crosslinking can be obtained [62]. Hashemnejad et al. [63] reported the failure behavior and nonlinear rheological characteristics of alginate hydrogels, a class of polysaccharide hydrogels made by covalent and ionic crosslinking. Ionic alginate hydrogels, also known as gels with ionic crosslinks, are created by adding Ca^2+^ ions to an aqueous solution of sodium alginate. Covalently crosslinked alginate gels, also known as chemical alginate hydrogels, are produced by amidation reactions. When exposed to large amplitude oscillatory shear (LAOS), both chemical and ionic alginate hydrogels show strain stiffening behavior, which is accompanied by negative normal stress. Even though the linear elastic modulus of ionic and chemical alginate hydrogels is similar, cavitation rheometry reveals distinct fracture or defect growth mechanisms.

Rheological properties of alginate gel were determined by using different types of rheometers and taking temperature into account. Since temperature parameters affect rheological data, the temperature of the storage and production areas should be considered to check the rheological properties of hydrogels. In order to determine the dynamic viscosity characteristics of hydrogels, they were analyzed by dynamic sweep frequency test at a predetermined angular frequency. In most cases, the storage modulus (G′), loss modulus (G′′), and loss factor (tanΔ = G′′/G′) values are used to express the results of the rheological measurements [64]. The storage modulus (G′) and loss modulus (G′′) are used to interpret the analysis results. In this case, G′ > G′′ indicates elastic solid hydrogel properties, while G′ < G′′ indicates viscous fluid properties [65]. Steady rheology is also important for measuring the viscosity of alginate hydrogels and the correlation between the shear stress and shear rate. Within a specific shear rate range (typically 0.001~100 s^−1^), shear stress tests of the flat plate geometry are used to determine steady rheological properties and viscosity [66].

Dynamic rheological measurements can be performed using strain, frequency, and temperature sweep measurements. Especially in the biomedical sector, the characteristics of alginate hydrogels differ based on their function. Frequency and temperature scanning tests are used, which is necessary considering the area where the hydrogel will be used. Gong et al. [67] found that hydrogels showed shear thinning properties due to changes in angular frequency, and there was a synergistic effect among hydrogel components. For composite hydrogels, it is considered important to determine the synergistic and antagonistic properties of the materials added to the formulation to determine the properties of hydrogels.

## 3. Fabrication of Alginate Composite Hydrogels

Although alginate hydrogel is widely used in the biomedical field, single alginate hydrogels still have many defects in biological applications, such as uncontrollable swelling, weak cell adhesion, and poor mechanical properties [68], due to their strong hydrophilicity, which severely impedes alginate hydrogels' advancement and use in tissue engineering. An efficient way to address these issues is to prepare composite hydrogel materials by combining alginate and other materials through physical blending, layer-by-layer assembly, or interpenetrating network technologies. This does more than just remove a single material’s flaws; it also provides the material with a host of exceptional new physical, chemical, and biological properties, so that it can meet the application requirements as a BTE scaffold [22,69]. Among the various composite hydrogels, two kinds of nanocomposite hydrogels and interpenetrating network composite hydrogels with good mechanical properties have attracted extensive attention.

### 3.1. Nanocomposite Hydrogels

Because of their exceptional mechanical qualities and the distinctiveness of the nanomaterials themselves, nanocomposite hydrogels have recently opened up new possibilities for the production of functional hydrogels. Compared to regular hydrogels, nanocomposite hydrogels are highly hydrated polymer networks with improved elasticity, strength, and heat resistance [70]. Usually, to produce hydrogels made of nanocomposite materials, it is necessary to combine nanoparticles and the polymer. Compared with particles, nanoparticles have a smaller size and can be more evenly distributed when embedded in a polymer matrix. Some materials with nanofiber and nanostructured surfaces, for example, have been used to study extracellular matrices that mimic cartilage. Human cartilage contains only a small number of chondrocytes, but the dense nanostructured extracellular matrix is rich in proteoglycan, collagen, and elastin fibers. Therefore, the application of nanomaterials has excellent mechanical and biomimetic properties in terms of their nanostructural properties. Common nanoparticles include hydroxyapatite, clay, cellulose, and nano-titanium dioxide and silica particles, which reinforce the hydrogel matrix when used as fillers. By using these nanocomposites, hydrogels perform better in procedures involving cartilage regeneration [71]. Tanpichai and associates [72] created hydrogels of cellulose nanocrystalline and PVA nanocomposites, demonstrating their favorable mechanical characteristics and potential for use in tissue engineering and biomedicine.

**Figure 3:**
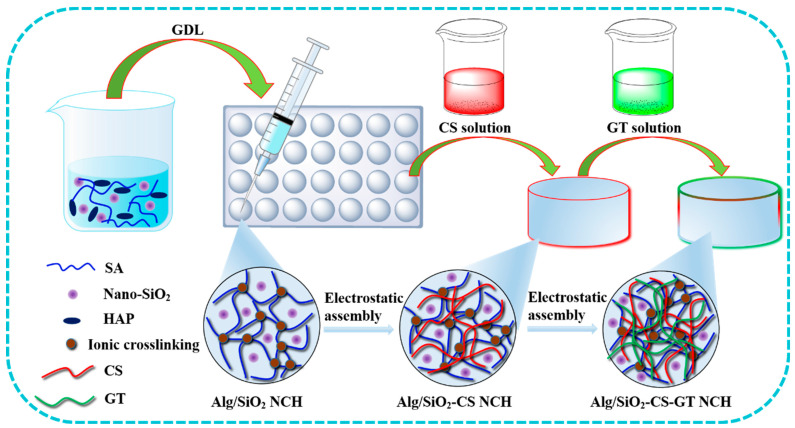
Diagram illustrating the process of creating Alg/SiO_2_-CHI-GA NCH using electrostatic adsorption and endogenous crosslinking [75].

With the development of biomaterials, nanocomposite hydrogels made of non-toxic, biodegradable, and biocompatible biopolymers are gaining popularity. Hyaluronic acid, chitosan, alginate, and other polysaccharides are becoming more and more important in this situation. Alginate nanocomposite hydrogels have been applied extensively for the regrowth of different organs and tissues, including the liver, pancreas, blood vessels, and cartilage [73,74]. Chen et al. [75] utilized gelatin (GA) and chitosan (CHI) in an alternate electrostatic adsorption procedure to combine Alg hydrogels with nano-silica (SiO_2_) to create nano-SiO_2_-reinforced Alg-chitosan-gelatin nanocomposite hydrogels (Alg/SiO_2_-CHI-GA NCH) for biomedical applications, as displayed in Figure 3. Studying biomineralization in vitro, it was found that the presence of Si-OH on the surface of nano-SiO_2_ enhanced NCH’s capacity for biomineralization. ALG/SiO_2_-CHI-GA NCH can promote 3D cell growth, and the in vitro cytocompatibility test revealed that adding nano-SiO_2_ can increase cell adhesion and the viability of MG63 and MC3T3-E1 cells. Alg/0.5% and Alg/1.0% SiO_2_-CHI-GA NCH, in particular, showed notable proliferative activity because of their uniform cell adhesion and high porosity. Nonetheless, the amount of nano-SiO_2_ added to the cells progressively raised the relative ALP activity of the cells, suggesting that nano-SiO_2_ promotes the osteogenic differentiation of MG63 and MC3T3-E1 cells.

### 3.2. Interpenetrating Network Composite Hydrogels

Interpenetrating polymer network (IPN) composite hydrogels are composed of composite hydrogels through interpenetrating and crosslinked entangling between two or more polymer molecular chains. In these IPN composite hydrogels, two or more polymer networks are independent of each other, but are intertwined by intermolecular chains. Fully interpenetrating polymer networks (IPNs) and semi-interpenetrating polymer networks (semi-IPNs) are the two categories into which they are separated based on the various crosslinking instances. In semi-IPN composite hydrogels, only one polymer is crosslinked, while the other polymer is interspersed and entangled in the cross-linked polymer [76]. Fully IPN composite hydrogels refer to network gels with a topological structure, which exist in the form of crosslinked networks penetrating each other. However, in IPN composite hydrogels, different polymer networks penetrate each other but are independent of each other, thus preserving each polymer’s distinct qualities and enhancing the composite hydrogels’ physical characteristics in the process [77]. Therefore, research on the composition and characteristics of composite hydrogels with interpenetrating networks is beneficial to expanding the uses of hydrogels in the biomedical industry.

**Figure 4:**
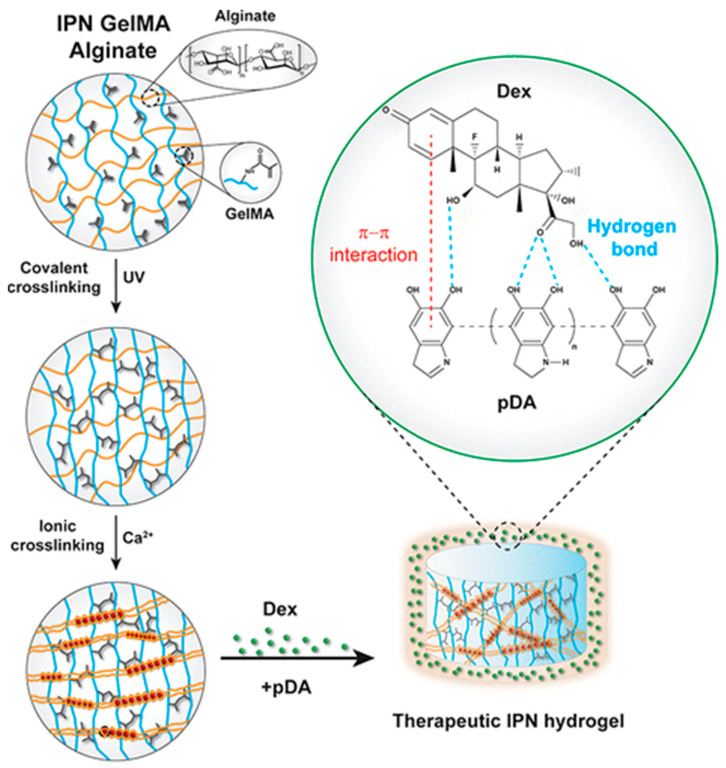
Mechanism diagram of an alginate/methylacrylylated gelatin IPN composite hydrogel [80].

Recently, alginate and natural polymers as well as their derivatives (polysaccharides and proteins) or synthetic polymers have been blended to create IPN composite hydrogels, which address the shortcomings of pure alginate hydrogels that have low biological activity and poor mechanical properties. For example, Hu et al. [78] demonstrated the use of a coaxial microfluidic chip to create stretchable helical hydrogel microsprings using an interpenetrating network (IPN) hydrogel made of alginate and GelMA materials. Helical structures are known for their superior elasticity. Furthermore, the hydrogel microsprings offered an appropriate mechanical microenvironment for improving cell infiltration and supporting in situ muscle tissue regeneration in rat models when they were sized appropriately and used as injectable micro-scaffolds. Bai et al. [79] prepared poly(N,N-dimethylacrylamide) (PDMAA)/alginate (SA) IPN composite hydrogels and PDMAA/chitosan (CS) semi-IPN composite hydrogels using alginate, N, N-dimethylacrylamide (DMAA), and chitosan as the raw materials. The mechanical strength and fatigue resistance of the two types of composite hydrogels were analyzed and studied, and it was discovered that their mechanical strength could be greatly increased by adding natural polymers, while PDMAA/SA IPN composite hydrogels showed higher mechanical strength but poor stability, and PDMAA/CS semi-IPN composite hydrogels showed excellent fatigue stability. However, their mechanical strength was relatively low, indicating that IPNs had a high crosslinking density conducive to toughening, while semi-IPNs were conducive to energy dissipation. Pacelli et al. [80] obtained IPN composite hydrogels of methylacrylylated gelatin (GelMA) and alginate based on the photochemical crosslinking of GelMA and ion crosslinking of alginate, and then modified the IPN composite hydrogels with polydopamine (pDA). A pDA adhesive layer is formed on their surface to adsorb the bone-inducing drug dexamethasone (Dex), as shown in Figure 4. In the first week of the experiment, the results demonstrated that Dex was effectively adsorbed and retained on the surface of the composite hydrogels by pDA, causing adipose-derived stem cells to undergo delayed osteogenic differentiation. To increase the composite hydrogels’ suitability for bone tissue regeneration, the dose relationship between Dex and pDA should be further optimized to realize the regulation of the adsorption retention time of Dex on the composite hydrogels by pDA, thus achieving the purpose of regulating the osteogenic differentiation of stem cells.

### 3.3. Three-Dimensional Bioprinting of Alginate-Based Bio-Ink

Tissue engineering scaffolds are created through 3D bioprinting by utilizing hydrogel inks infused with particular cells. High-throughput fabrication, high spatiotemporal resolution, and the ability to place desired micro/nanostructures at the target site in the scaffolds are some of the benefits that 3D bioprinting offers over traditional methods [81]. The 3D bioprinting technique has witnessed a notable enhancement in printing speed, resolution, and accuracy of 3D-printed scaffolds due to the rapid advancements in mechanical instrumentation and software technology [66]. Hydrogel inks based on alginate have shown great promise in creating 3D bioprinted scaffolds that mimic the native tissue microenvironment and have tunable degradability and non-toxic by-products. Alginate is a naturally occurring biocompatible polymer that is widely used in the 3D printing of cartilage and bone. It is an excellent option for hydrogel ink material because it is a natural polymer that is not derived from animals [77]. Lately, Monavari et al. [82] provided an example of the application of bioactive glass nanoparticles (mesoporous silica–calcia nanoparticles, or MSNs) to reinforce alginate dialdehyde–gelatin (ADA-GEL) hydrogels. The hydrogel bio-ink’s mechanical properties significantly improved with the addition of silica nanoparticles. Utilizing the same nanoparticles, phytotherapeutic icariin—known for its osteogenic effects—was also administered. Yang et al. [39] found that, when compared to pure alginate and alginate–agarose bio-inks, combining alginate with collagen, a naturally occurring form of gelatin, increased chondrocyte proliferation and survival as well as matrix (GAG) production. Compared to the alginate and alginate–agarose constructs, the alginate–collagen 3D-printed construct had a significantly higher cell viability after 14 days of culture. Yang et al. [83] attempted to create hepatorganoids (3DP-HOs) using alginate-based bio-ink made of gelatin and alginate, as well as the 3D bioprinting of HepaRG cells to create a liver tissue model. Then, both in vitro and in vivo liver functions were examined for the artificial hepatorganoid.

## 4. Biomedical Application of Alginate Composite Hydrogel in BTE

Over the years, due to the increase in the number of patients with bone diseases caused by trauma, bone tumors, congenital bone defects, sports accidents, traffic accidents, and joint pain, autologous transplantation, allotransplantation, and other treatment methods are usually used to solve these bone diseases, but these treatment methods are limited by their own defects [4,5]. BTE is a new method to promote bone repair and regeneration and restore its function, which is considered to be able to make up for the deficiencies of autologous bone transplantation and allograft bone transplantation [84]. BTE aims to repair the defect of bone and restore its function, and then develop and prepare artificial scaffolds with structure, function, and mechanical properties equivalent to healthy bone. As a BTE scaffold, it must also have an interconnected and porous pore structure to facilitate the attachment, proliferation, and differentiation of osteoblasts, which should be beneficial to the growth and functional recovery of bone tissue. To provide a channel for the transport of nutrients and the discharge of metabolic waste, BTE scaffolds must also have sufficient mechanical strength to provide support for the regeneration of bone tissue at the implant site, and should also be appropriately biodegradable and biocompatible, non-toxic, and promote good integration with the tissues around the implant site [25].

The application of alginate in BTE has been widely reported [24,26,27,85,86], and alginate polysaccharides have been certified by the US FDA as safe for human use [23]. Meanwhile, alginate hydrogels prepared by Ca^2+^ crosslinking have reportedly been widely studied in biomedical fields, as presented in Table 1, including scaffolds and carriers of bioactive molecules or cells in cartilage and bone tissue regeneration applications, which have been shown to promote the regeneration of new cartilage and bone [87,88,89]. Compared with traditional surgery, they can be injected into the affected area of the patient in a minimally invasive way to achieve the purpose of treating or repairing the defective bone, and they easily undergo chemical modification and can control the release of tissue-inducing factors, such as BMP and TGF-β [90,91]. Secondly, alginate hydrogels also have structural characteristics similar to the human extracellular matrix [92,93]. Based on these advantages, alginate hydrogels have a promising future in the field of tissue engineering, as displayed in Table 2.

However, pure alginate-based porous hydrogels still have certain drawbacks, including low mechanical strength [12,16], a deficiency in binding sites specific to individual cells [106,107], a propensity for hydrogel structure disintegration in a physiological setting [108], and low degradation of alginate hydrogels in vivo, which severely limits their application in BTE [26]. For this reason, researchers have combined alginate with other substances, such as biomacromolecules, synthetic organic polymers, biological proteins, and Arg-Gly-Asp (RGD) peptides, bioceramics, bioglass, and nanoclay, using the respective advantages of these substances to enhance the shortcomings of pure alginate hydrogels used as BTE scaffold materials. The use of complementary advantages to achieve a win–win outcome makes it possible for alginate hydrogel scaffolds to be applied in clinics at an early date, and promotes regenerative composite biomaterials, which are expected to replace traditional autologous transplantation and allotransplantation in the future [109].

### 4.1. Alginate/Polymer Composite Hydrogels

**Figure 5:**
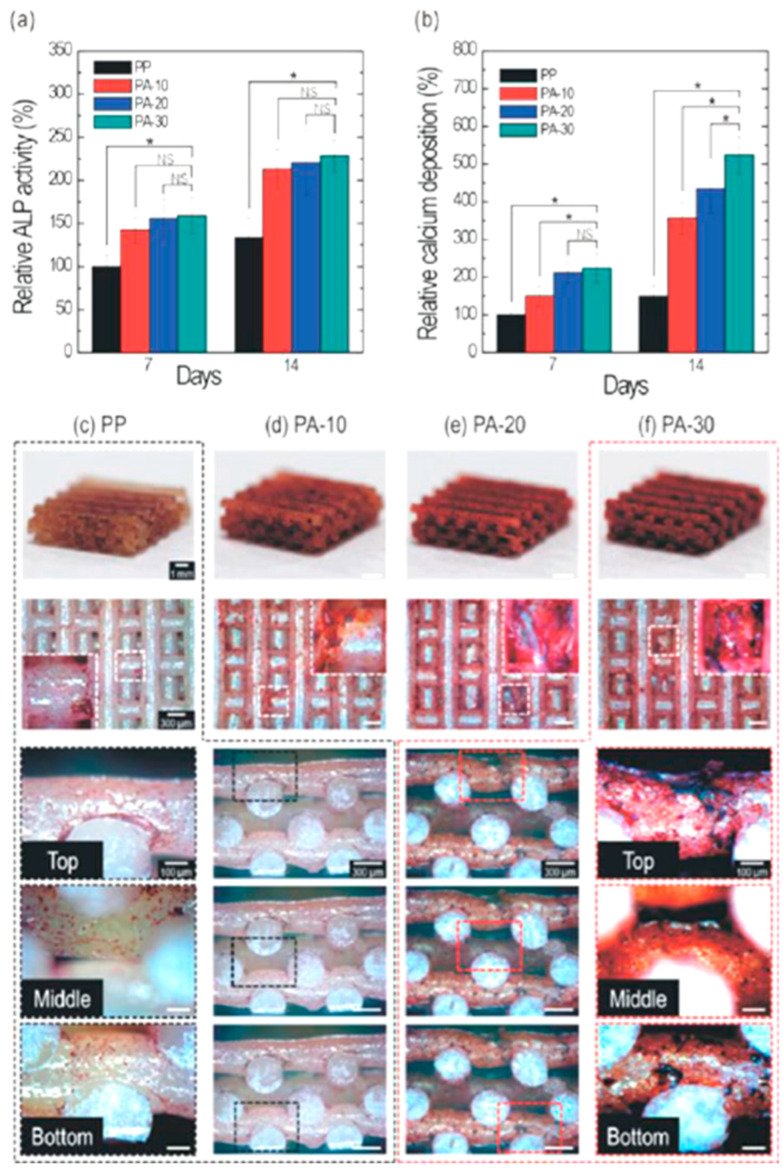
On composite scaffolds with different alginate weight fractions from 7 to 14 days, (**a**) relative alkaline phosphatase (ALP) activities and (**b**) relative calcium deposition levels were measured. (**c**–**f**) Three-dimensional form and cross-sectional optical pictures of the scaffolds’ ARS staining following a 14-day culture period. A significant difference is indicated by * *p* < 0.05, while non-significance is indicated by NS [115].

Due to the lack of mechanical properties matching natural bone, the development of alginate hydrogels in BTE is limited, so some researchers compound them with some synthetic organic polymers, such as polylactic acid–glycolic acid (PLGA), polycaprolactone (PCL) [110,111,112], or with some natural polymers, such as bacterial cellulose (BC) and cellulose nanocrystals (CNCs) [113,114]. In the field of biomedicine, the produced composite hydrogel has demonstrated a strong application potential. Blending natural biomacromolecules or synthetic polymers can enhance the mechanical strength or biological activity of alginate hydrogels, so as to provide mechanical support for bone regeneration. For example, polycaprolactone (PCL) is a kind of synthetic organic polymer with good biocompatibility and biodegradation, and is one of the polymer materials approved by the US FDA and can used in surgery. It has a great development prospect in BTE, but it has shortcomings, such as strong hydrophobicity and a lack of biological activity. Kim et al. [115] designed composite scaffolds based on PCL and alginate to enhance the mechanical characteristics of alginates. With different weight fractions of alginate (10, 20, and 30% wt.%), the PCL/alginate composite scaffolds showed distinct pore structures made up of micro-sized struts layered one on top of the other. A considerable improvement in water absorption and wetting behavior was noted, despite the PCL/alginate composite scaffolds’ lower Young’s modulus when compared to the pure PCL scaffold. In terms of biology, on composite scaffolds composed of different weight fractions of alginate, preosteoblast (MC3T3-E1) cells were cultivated. Relative to the pure PCL scaffold, there was a noticeable improvement in both cell viability and cell-seeding efficiency. Following a 14-day culture period, optical imaging of ARS staining provided quantitative confirmation that PCL/alginate composite scaffolds, as opposed to pure PCL scaffolds, significantly induced cell differentiation, as demonstrated by ALP and mineralization assays, as presented in Figure 5. Quinlan et al. [116] explored the creation of collagen–hydroxyapatite scaffolds with alginate and PLGA microparticles for the localized and continuous delivery of rhBMP-2. Both sets of microparticles within the scaffolds effectively released bioactive rhBMP-2; however, in vitro, the growth factor delivered by the PLGA-collagen hydroxyapatite scaffolds was more pro-osteogenic than that of the alginate–collagen hydroxyapatite scaffolds. After eight weeks of implantation in a critical-sized bone defect, the regenerative effect was demonstrated by the significantly higher bone regeneration caused by PLGA-collagen hydroxyapatite scaffolds. Zhu et al. [117] created alginate/bacterial cellulose–chitosan (Alg/BC-CS) composite scaffolds by covering the alginate matrix’s surface with CS and incorporating BC into it. The gelling system used in this process was the hydroxyapatite/D-glucono-d-lactone (HAP/GDL) complex. The mechanical strength of the Alg/BC-CS composite scaffolds was found to increase from 0.132 to 0.269 Mpa, as demonstrated by the experimental results, which also showed a well-developed pore structure with an average size of 110 mm and regular 3D morphology. Inhibiting the breakdown of Alg/BC-CS composite scaffolds and effectively controlling swelling behaviors are made possible by BC’s tight fiber network structure, strong barrier qualities, and ability to form intermolecular hydrogen bonds with alginate. Together with good cytocompatibility, they also demonstrated good protein adsorption and release performance and the ability to form apatite. Due to their advantages, Alg/BC-CS composite scaffolds have the potential to be used in tissue engineering for biomedical purposes. It can be seen from the above discussion that the addition of synthetic organic polymers or natural biomacromolecules can improve the mechanical properties or enhance the biological activities of alginate hydrogel scaffolds.

### 4.2. Alginate/Bioprotein or Polypeptide Composite Hydrogels

The use and advancement of pure alginate hydrogels in BTE scaffolds have been constrained by their lack of cell-specific recognition sites. Therefore, some scholars have combined alginate with some biological proteins, such as collagen [113], bovine serum albumin [118], and polypeptides, such as Arg-Gly-Asp (RGD), and the prepared alginate composite hydrogels have shown good application prospects in the biomedical field.

**Figure 6:**
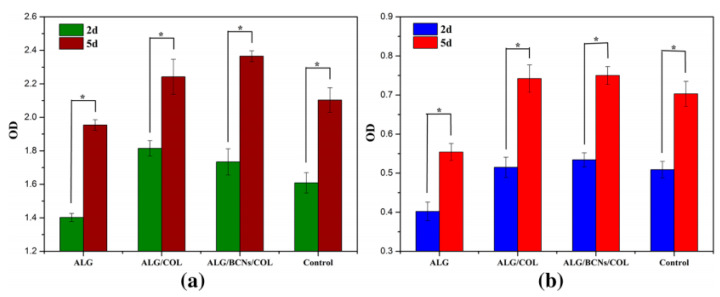
(**a**) MC3T3-E1 and (**b**) h-AMS cells were used in a CCK-8 assay to assess the cell viability of the composite scaffolds. * *p* < 0.05 [113].

It is report that alginate and some bioactive proteins or polypeptides (RGDs) can be compounded by physical or chemical modifications to provide the alginate hydrogel scaffold with cell-specific recognition sites, so that osteoblasts can adhere, proliferate, and differentiate on its surface, thus making it a hopeful candidate for BTE applications. Yan et al. [113] combined alginate with collagen (COL) and bacterial cellulose (BCN) to fabricate homogeneous ALG/BCNs/COL composite hydrogel scaffolds through ion crosslinking under the condition of CaCO_3_-GDL. The results showed that the adhesion of MC3T3-E1 cells and h-AMS cells was poor on pure alginate hydrogel scaffolds, but good on alginate composite hydrogel scaffolds mixed with collagen, indicating that the ALG/BCNs/COL composite hydrogel scaffolds’ biological activity was noticeably higher than that of the hydrogel material made entirely of alginate. In vitro cell culture experiments on ALG/COL and ALG/BCNs/COL composite hydrogels demonstrated good cell growth and proliferation for both MC3T3-E1 and h-AMS cells, indicating that ALG/BCNs/COL composite hydrogels had good biocompatibility, as shown in Figure 6. In addition, using measurements of the composite hydrogels’ mechanical characteristics, porosity, and swelling rate proved that the composite scaffolds have an appropriate swelling property, high porosity, good compressive strength, and excellent biocompatibility, making them ideal scaffold materials with potential application value. Yao et al. [119] chemically modified alginate with an RGD peptide and successfully prepared a microporous alginate composite hydrogel (RGD/MA). In an in vitro cell culture experiment, ATDC5 cells were inoculated on a microporous RGD/MA scaffold and control group of an MA scaffold, and a CCK-8 kit was used to measure the ATDC5 cells’ ability to proliferate on MA and RGD/MA scaffolds. It was found that the cells in both groups showed good proliferation trends during cell culture. However, compared with the control group of the MA scaffold, the RGD/MA scaffold could promote cell proliferation, indicating that alginate can improve the biological activity of alginate hydrogels after the chemical modification of RGD. Then, a series of experiments including RT-PCR, hematoxylin eosin (H&E) and Toluidine blue O staining cells proved that the RGD/MA composite scaffold could improve the expression of cartilage genes, indicating that the biocompatibility of alginate composite hydrogel scaffolds was improved by RGD modification.

### 4.3. Alginate/Bioceramic Composite Hydrogels

**Figure 7:**
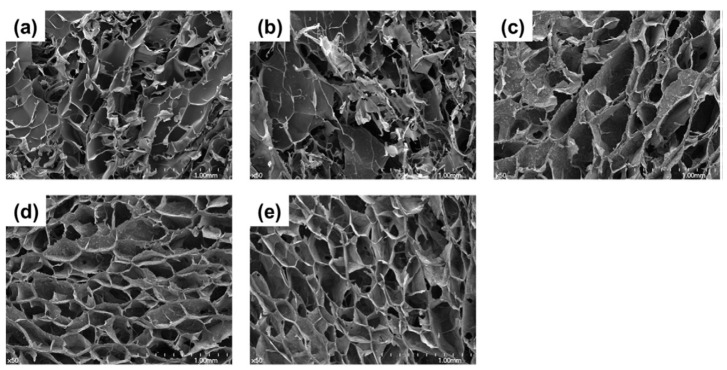
SEM images of the microstructures of cross-sectional scaffolds made of alginate and chitosan composites with different concentrations of HAP. The composite scaffolds’ nano-HAP contents were (**a**) 0 wt.%, (**b**) 10 wt.%, (**c**) 30 wt.%, (**d**) 50 wt.%, and (**e**) 70 wt.%. A total of 50× magnifications were used initially [123].

Bioceramics can be used as bone replacement materials in the field of BTE, and the research on bioceramics focuses on hydroxyapatite (HAP), calcium phosphate (TCP), and their mixture (BCP) [120]. Bioceramics are inorganic substances, mainly composed of calcium and phosphorus ions, which have an inorganic composition and crystal structure similar to natural bone; so, bioceramics have good biocompatibility, bone conductivity, and bone induction abilities, and also have a high Young’s modulus, and are widely used in biomedical fields [121]. Therefore, the bone conductibility, mechanical properties, and biocompatibility of composite hydrogels can be significantly improved by incorporating bioceramics into alginate hydrogels. Liu et al. [122] developed a bioactive SA/HAP porous hydrogel scaffold by mixing SA with different amounts of nano-hydroxyapatite (HAP), under the action of GDL for Ca^2+^ crosslinking. Finally, a number of hydrogel scaffolds made of SA/HAP composite were effectively constructed, and their mechanical qualities were examined. The findings reveal that the stress of the 7 wt.% HAP porous composite hydrogel is 8.5-times that of the 1 wt.% HAP porous composite hydrogel. This suggests that the addition of HAP can enhance the mechanical strength of the composite hydrogel and improve the mechanical strength of the porous alginate fiber scaffold. Apatite particles formed on the surface of the porous composite hydrogel scaffold after the SA/HAP composite hydrogel scaffold was submerged in simulated body fluid (SBF) for mineralization, suggesting that the scaffold has good biomaterial activity. Additionally, the composite hydrogel scaffold was seeded with mouse bone marrow mesenchymal stem cells (mBMSCs) for in vitro cell culture, and it was observed that mBMSCs could adhere and proliferated well on the composite hydrogel scaffold, providing evidence of the high biocompatibility of the SA/HAP composite hydrogel scaffold. Kim et al. [123] dispersed nano-HAP in an alginate and chitosan solution and prepared composite scaffold with the freeze-drying method. The composite hydrogels exhibited a significant increase in compressive strength and elastic modulus from 0.27 MPa and 4.42 MPa to 0.68 MPa and 13.35 MPa, respectively, as the HAP content rose to 70 wt.%. The composite hydrogels’ pore structure was unaffected by the addition of HAP; instead, the pore size and pore channel became more uniform, as shown in Figure 7. HAP may also encourage the composite scaffold’s cell adhesion, proliferation, and differentiation. In addition, TCP is very similar to human bone and has been widely used in clinical practice. In particular, it is mixed with organic polymers like chitosan and alginate, which has a potential application value in tissue engineering [124].

### 4.4. Alginate/Bioglass Composite Hydrogels

Bioglass is a kind of inorganic material with good biological activity, mainly composed of oxides of sodium, calcium, phosphorus, and silicon. Currently reported bioglass include compounds composed of silicate or phosphate system, 45S5 bioglass^®^, 58S bioglass, silica, and titanium dioxide [125,126,127]. Bioglass has been widely reported to be applied to BTE, and it has shown good biological activity and bone binding ability in the field of BTE. Adding bioglass to an alginate matrix can significantly improve cell proliferation rates and osteogenic differentiation [128].

**Figure 8:**
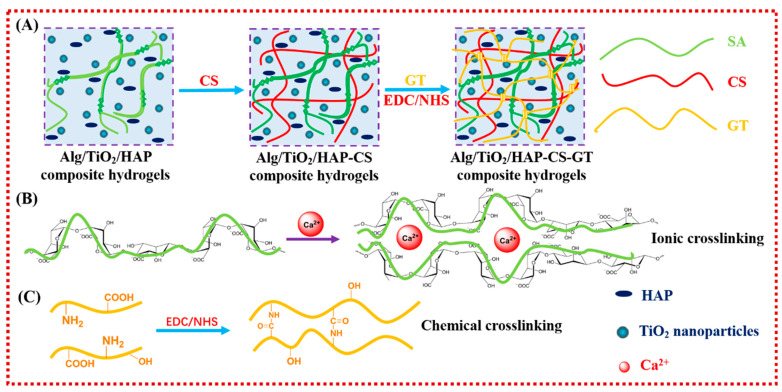
(**A**–**C**) Diagram showing the sequential electrostatic assembly of CS and GT after the endogenous ionic crosslinking of alginate to create Alg/TiO_2_/HAP-CS-GT composite hydrogels [130].

In recent years, some scholars have prepared scaffold materials by combining alginate with bioglass to make up for the defects of alginate hydrogel itself. Ye et al. [129] prepared a bioglass/gelatin/sodium alginate (BG/Gel/SA) composite scaffold. The addition of more bioglass to the BG/Gel/SA composite scaffold greatly enhanced its compressive strength and capacity for biomineralization, but the compressive strength of the BG/Gel/SA composite scaffold rapidly decreased when the weight percentage reached up to 30%. The results of in vitro cell culture experiments demonstrate that the BG/Gel/SA composite scaffold has good biocompatibility, as evidenced by the well-attached cells on its surface. Additionally, as the amount of bioglass increased, rat bone marrow mesenchymal stem cells (mBMSCs) on the BG/Gel/SA composite scaffold proliferated and underwent osteogenic differentiation. These findings suggest that bioglass may enhance the biological activity of alginate scaffolds. It is evident that the biological activity and mechanical characteristics of alginate composite scaffold material may be enhanced by the addition of bioglass, providing it with a potential application value in BTE. Chen et al. [130] tried to create titania/hydroxyapatite-promoted biomimetic alginate–chitosan–gelatin (Alg/TiO_2_/HAP-CS-GT) composite hydrogels by physically blending HAP nanoparticles as the endogenous crosslinking agent and reinforcing agent, then altering the surface by electrostatically assembling chitosan (CS) and gelatin (GT) in alternate cycles to fundamentally improve the bio-applicability of alginate hydrogels (as shown in Figure 8). The produced Alg/TiO_2_/HAP-CS-GT composite hydrogels had a porous structure and good three-dimensional morphology. TiO_2_ nanoparticles can be added to composite hydrogels to effectively control their pore structure, mechanical characteristics, swelling, in vitro biodegradation, and biomineralization. An in vitro cytotoxicity test revealed that the pore wall and exterior surface of the ALG/TiO_2_/HAP-CS-GT composite hydrogels were suitable for MC3T3-E1 cell adhesion and dissemination. Furthermore, the addition of TiO_2_ nanoparticles could effectively promote cell differentiation, as evidenced by the gradual increase in the relative ALP activity of cells on the Alg/TiO_2_/HAP-CS-GT composite hydrogels with the enhancement of TiO_2_ content.

### 4.5. Alginate/Nanoclay Composite Hydrogels

Nanoclay is a layered silicate material with a charge distributed on the interlayer surface and large specific surface area. It can promote the interaction between cells and biomacromolecules through an electrostatic action, and it can also induce the transmission of biological signals in cells, so it has great application potential in the field of BTE [131,132,133]. Wei et al. [134] successfully prepared a composite hydrogel scaffold by adding sodium alginate (SA), EDTA-Ca, and TEMPO oxidized bacterial cellulose (TOBC) into the nanoclay (Xls) solution under the crosslinking of a GDL and CaCl_2_ solution. The outcomes demonstrated a significant impact on the cell proliferation of the composite hydrogel with an Xls content less than 0.5 wt.%, so the appropriate addition of nanoclay could enhance the bioactivity and mechanical properties of alginate composite hydrogel scaffolds. Hakimi et al. [135] created innovative layer-by-layer electrospinning scaffolds for chitosan, polyethylene oxide, and nanoclay alginate (CS-PEO/NC-ALG). Good cell biocompatibility and hemocompatibility were demonstrated by the CS-PEO/NC-ALG scaffolds. As determined by FE-SEM and XRD methods, the mineralization process produced bone-like apatite. The incorporation of nanofiber and nanoclay into CS-PEO/NC-ALG scaffolds improved their mechanical characteristics and mineralization potential. CS-PEO/NC-ALG may be utilized for bone tissue regeneration, according to additional analysis that takes into account swelling, biodegradability, and mineralization assays.

## 5. Current Status and Challenges

At present, the method of Ca^2+^ exogenous crosslinking is often applied to prepare alginate hydrogels at home and abroad. Its primary method of crosslinking depends on Ca^2+^ infiltration, which can easily result in flaws like uneven crosslinking and scaffold deformation. This not only diminishes the hydrogel’s mechanical integrity and strength, but also decreases its plasticity. Even though the internal crosslinking of Ca^2+^ is used to achieve homogeneous crosslinking, the residual problem of insoluble calcium salts is rarely considered. Since the appropriate microscopic pore structure is the key to the optimal osteogenic performance of BTE porous scaffolds, the regulation of the crosslinking density in homogeneous hydrogels to regulate the pore size and porosity of hydrogel materials was seldom taken into consideration by our predecessors. Secondly, the effect of alginate crosslinking structure on the degradation and surface activity of scaffolds is reported minimally. In order to attain a balance between the mechanical characteristics of the scaffold material and its rate of degradation, it is imperative to investigate the hydrogel’s degradation mechanism in normal saline as well as the adhesion properties of cells on its surface by merging the hydrogel’s structural and mechanical properties. Finally, the impact of the composite hydrogel’s controlled release of certain active biological growth factors (like BMP and TGF-β) on the differentiation and proliferation of cells on the scaffold material, and the optimal design of the morphology and structure of the interconnected composite hydrogel must be considered. Only by analyzing and solving these basic research problems of structure and properties can the clinical application of alginate composite hydrogels be improved fundamentally.

## 6. Conclusions and Future Perspectives

The objective of the present work is to use renewable alginate with large reserves and good biocompatibility to develop composite hydrogels with a uniform texture, regular structure, controllable degradation performance, and suitable mechanical strength, surface adhesion, recognition, and induction characteristics, so as to solve the defects of alginate in BTE applications, thus providing a theoretical basis for the development and sustainable application of alginate-based functional biomedical materials. Alginate’s excellent biocompatibility, biodegradability, lack of immunogenicity, abundant source, and affordable cost have made it a popular choice for BTE. Although alginate hydrogels have a potential application value in the field of BTE, compared with natural bone, pure alginate hydrogel scaffolds still have defects, such as insufficient mechanical properties and low biological activity (that is, a lack of mammalian specific recognition sites). It is these problems that make it difficult to achieve clinical applications for alginate composite hydrogel scaffolds. To overcome these defects, researchers consider the advantages of natural polymers or synthetic organic polymers, biological proteins or peptides, bioceramics, bioglass, nanoclay, and other materials to make up for the shortage of alginate hydrogels, and use composite methods to improve the bioapplicability of alginate hydrogels. Therefore, it is anticipated that alginate composite hydrogel scaffolds can become an ideal BTE scaffold material after making up for their shortcomings, so as to repair or treat bone defects, fractures, and other problems, shed light on patients with bone diseases, and aid in raising people’s standard of living and health.

Notably, rather than offering a thorough examination of and solution for the structural- and property-based functional limitations of alginate hydrogels in biomedicine, the majority of previous studies have only focused on the drawbacks of these hydrogels alone. It is specifically unclear how the macro-properties of hydrogels and the nanostructure of polymer molecules relate to one another. Therefore, the future research direction should concentrate on conducting thorough structural and property analyses of alginate defects in tissue engineering. The unique properties of composite hydrogels should be used to address the shortcomings of alginate hydrogels in a fundamental way, thereby enhancing their bioapplicability. The extensive study of BTE scaffolds based on alginate composite hydrogels has practical guiding significance for the development and sustainable application of natural renewable materials.

## Figures and Tables

**Table 1:** Alginate hydrogel-based materials for biomedical applications.

Alginate Hydrogel-Based Materials	Representative Property	Biomedical Application	References
Alginate-g-poly(N-vinyl imidazole)	Reduced rate of degradation and enhanced antimicrobial activity	Antimicrobial materials	[94]
Alginate/gelatin/nano-apatite	Proliferation and osteogenic differentiation	Hydrogel scaffold	[95]
Alginate/chitosan/collagen composite	Excellent mechanical qualities, good biosecurity, and good water absorption	Full-thickness wound healing	[96]
Alginate/ZnO	Antibacterial, enhanced wound healing	Bi-layered hydrogel dressings	[97]
Alginate/gelatin/polyvinyl alcohol	Absorb exudates, maintain a moist environment, and enhance the interaction with the tissues	Wound dressing	[98]
Alginate/chondroitin sulfate	Enhanced tissue regeneration capacity	Diabetic wound healing	[99]
Alginate/lecithin (loading peppermint oil)	Excellent swelling and muco-adhesion qualities	Oral delivery	[100]
Alginate dialdehyde/gelatin/fibrin	Increased potential for cell adhesion and growth	Liver tissue engineering	[101]
Alginate/bioactive glass composite hydrogels	Enhanced mechanical, bioadhesion, and osteogenic differentiation properties	Bone tissue engineering	[102]

**Table 2:** Composites made of alginate for use in tissue engineering.

Alginate-Based Composites	Representative Property	Tissue Engineering Application	Reference
Alginate/collagen	Superior cell adhesion and proliferation, strong mechanical strength, and increased expression of genes specific to cartilage	Cartilage tissue engineering	[39]
Alginate/bioactive glass composite hydrogels	Improved osteogenic differentiation, mechanical, and bioadhesion properties	Bone tissue engineering	[102]
Alginate/chitosan/flurbiprofen	Excellent mechanical qualities, anti-inflammatory and hydrophilic qualities	Skin tissue engineering	[103]
Alginate/polycaprolactone/carboxymethyl chitosan	Excellent osteoinductive capacity, biocompatibility, and mechanical qualities	Periosteal tissue engineering	[104]
Alginate/Ga-based glass	Enhanced mechanical properties and biocompatibility	Cardiovascular tissue engineering	[105]

## Data Availability

The data presented in this study are available on request from the corresponding authors.

## Abbreviations

Bone tissue engineering	BTE
Hydroxyapatite	HAP
Polyethylene glycol	PEG
Polyvinyl alcohol	PVA
Polylactic acid	PLA
Polyacrylamide	PAM
Polylactic acid–glycolic acid	PLGA
Polycaprolactone	PCL
Food and Drug Administration	FDA
Three-dimensional	3D
Arg–Gly–Asp	RGD
Cell Counting Kit-8	CCK-8
Alkaline phosphatase	ALP
Polyelectrolyte complexes	PECs
Tetrazine modified alginate	Alg-T
Norbornene modified alginate	Alg-N
Large amplitude oscillatory shear	LAOS
Storage modulus	G′
Loss modulus	G″
Alg-chitosan-gelatin nanocomposite hydrogel	Alg/SiO_2_-CHI-GA NCH
Interpenetrating polymer networks	IPNs
Poly(N,N-dimethylacrylamide)	PDMAA
Chitosan	CS
Methylacrylylated gelatin	GelMA
Polydopamine	pDA
Dexamethasone	Dex
Alginate dialdehyde–gelatin	ADA-GEL
Hepatorganoids	3DP-HOs
Arg-Gly-Asp	RGD
Bacterial cellulose	BC
Cellulose nanocrystals	CNCs
D-glucono-d-lactone	GDL
Collagen	COL
Bone marrow mesenchymal stem cells	mBMSCs
Bioglass/gelatin/sodium alginate	BG/Gel/SA
Titania/hydroxyapatite-promoted biomimetic alginate-chitosan–gelatin	Alg/TiO_2_/HAP-CS-GT
TEMPO oxidized bacterial cellulose	TOBC
Chitosan–polyethylene oxide/nanoclay–alginate	CS-PEO/NC-ALG
